# Turning and Radius Deviation Correction for a Hexapod Walking Robot Based on an Ant-Inspired Sensory Strategy

**DOI:** 10.3390/s17122710

**Published:** 2017-11-23

**Authors:** Yaguang Zhu, Tong Guo, Qiong Liu, Qianwei Zhu, Xiangmo Zhao, Bo Jin

**Affiliations:** 1 Key Laboratory of Road Construction Technology and Equipment of MOE, Chang’an University, Xi’an 710064, China; guotong@chd.edu.cn (T.G.); liuqiong@chd.edu.cn (Q.L.); zhuqianwei@chd.edu.cn (Q.Z.); 2 State Key Laboratory of Fluid Power and Mechatronic Systems, Zhejiang University, Hangzhou 310028, China; bjin@zju.edu.cn; 3 School of information Engineering, Chang’an University, Xi’an 710064, China; xmzhao@chd.edu.cn

**Keywords:** ant-inspired, arbitrary gait, hexapod robot, turning, radius correction

## Abstract

In order to find a common approach to plan the turning of a bio-inspired hexapod robot, a locomotion strategy for turning and deviation correction of a hexapod walking robot based on the biological behavior and sensory strategy of ants. A series of experiments using ants were carried out where the gait and the movement form of ants was studied. Taking the results of the ant experiments as inspiration by imitating the behavior of ants during turning, an extended turning algorithm based on arbitrary gait was proposed. Furthermore, after the observation of the radius adjustment of ants during turning, a radius correction algorithm based on the arbitrary gait of the hexapod robot was raised. The radius correction surface function was generated by fitting the correction data, which made it possible for the robot to move in an outdoor environment without the positioning system and environment model. The proposed algorithm was verified on the hexapod robot experimental platform. The turning and radius correction experiment of the robot with several gaits were carried out. The results indicated that the robot could follow the ideal radius and maintain stability, and the proposed ant-inspired turning strategy could easily make free turns with an arbitrary gait.

## 1. Introduction

Compared with wheeled robots [1] and tracked robots [2], the movement of multi-legged robots is achieved by the alternating motion of each leg where the contact between the robot and ground is a series of discrete points. These unique advantages can allow them to cross small obstacles and give them the ability to move across uneven terrains [3]. It also has the ability of fault tolerance [4]. When one leg is broken or has failed, the rest of the legs will complete the operation of the robot continuously. Furthermore, a multi-legged robot has the same running or galloping potential as animals [5], which greatly expands its application range. Therefore, at present, the advantages of multi-legged robots have attracted many scholars to this field.

For a legged robot, one key point is to reasonably plan the desired movement of each leg to achieve smooth locomotion. The turning gait has strong expansibility, and can produce abundant movement forms. When the radius is sufficiently large, the turning path can be regarded as a straight line [6]. Fortunately, any possible path can be supposed as arcs and lines. Hence, the study of turning is an important part of legged robots. Some scholars have proposed a turning algorithm based on a single gait [7,8] and precise motion control [9,10]. According to the relative motion between foot tip and body, the function of foot tip was obtained to make the robot follow the desired path. However, this algorithm only can make the robot turn with a specific gait and does not contribute to the adaption of different terrains.

Some scholars have also planned robot movements based on biological inspiration [11,12,13]. Through evolution, creatures have formed skillful mechanism structures and nimble movement patterns. They are rational, scientific, and progressive in structure, performance, information processing, and autonomic learning. The study of bio-inspired robots includes the imitation of structure [14], motion principle [15], and behavior pattern [16]. Currently, the structure of bio-inspired robots imitates the structure of mammals or insects, and most of these structures have a great load capacity or flexible movement performance. The movement and behavior of creatures have provided the basis for robot design in areas such as gait planning, motion control, and attitude adjustment. Inspired by the turning of ants, a turning algorithm of a hexapod robot based on a neural network was proposed by Iwase et al. [17]. As a result, smooth and stable turning in different curvatures was achieved. To obtain the motion of each cockroach leg, Jindrich et al. [18,19] used a photoelastic technique to calculate the force of each cockroach leg during the movement and recorded it via a high-speed camera. The turning gait was studied according to the distribution of the stress point. The results indicated that the main power for turning was provided by the outboard legs, and the inboard legs provided surplus power. This experiment provided a theoretical basis for the dynamic analysis of hexapod robot turning. Fault-tolerant crab gaits and turning gaits were also raised [20,21]. On this basis, the omnidirectional walking of legged robots with a failed leg was proposed by the authors at the same time. This strategy improved the adaptability of the legged robot to the harsh environment to some extent. Similarly, turning can be controlled by a Central Pattern Generator (CPG) [22,23,24], and the output signal of the CPG was used to drive the robot, and the movement forms could be adjusted by the CPG parameters. To be more specific, the trajectory of the foot tip was a combination of three functions that controlled three directions. The input signal of each function was provided by three CPG signals. As long as the three CPG signals were controlled, different trajectories could be generated. This algorithm allowed the robot to turn in different directions. These methods have many advantages including easy control and multiple movement forms. Furthermore, some scholars have employed a chaotic neural oscillator as the central pattern generator (CPG) [25,26,27], which was considered as input for the inverse kinematic task, which provides particular trajectories. The coordinates of the foot tip are influenced by the turning radius. Each leg has to move along an arc of a circle and these arcs have the same center at the turn point, which is located on a line of the middle legs.

Most research into turning only considers a single gait and foot trajectory generation. However, the path tracking ability during turning is seldom analyzed. In other words, when the robot deviates from the desired path, it has no ability to correct and will even lead to errors. In fact, the robot will deviate from a desired path for a variety of reasons. Moreover, during omnidirectional movement, the robot needs to transform between various gait patterns and paths, which is adjusted by the motion parameters. Although the omnidirectional movement of robots has been studied, a detailed transition strategy among the different movements has not been discussed seriously.

Therefore, the ability to ensure stability in the transition process has become a hot topic. The main contribution with respect to the state-of-the-art in this paper was to solve the problems above-mentioned. A linear signal was the signal used as input for the movement of the robot, which avoided solving the CPG function and improved the efficiency of the program. An ant-inspired extended turning algorithm for a hexapod robot based on arbitrary gaits was proposed based on biological behavior. On the basis of the ant movement experiments in path correction, an ant-inspired radius correction algorithm was proposed, and a correction surface function was established without a complex environment model and positioning system. Finally, a bio-inspired hexapod robot platform was established. Furthermore, comparison experiments of radius transition, radius correction, and turning were conducted. Experimental results showed that the ant-inspired radius correction strategy and turning control algorithm were effective and had outstanding performance on stability.

## 2. Methods

### 2.1. Locomotion of Ant and Bio-Inspiration

Insects are a group of animals with a large number and type of species. Among all creatures (including bacteria, fungi, and viruses), insect species account for more than 50% of the total. The distribution of insects is so wide that no other creature can compare to them. Furthermore, the structural diversity and physiology of insects enables them to adapt and survive under a variety of environmental conditions, which has allowed them to prosper and remain powerfully competitive [28]. One of the most important reasons that insects have survived for millions of years is that they have flexible movement abilities [29], which have provided inspiration to the exploration of legged robots [30,31]. Most insects have a large number of legs which are symmetrically distributed on both sides of body, therefore, arranging each of leg allows them to cross all kinds of terrains [32].

The movement of an ant is closely related to the coordination of its legs. It can choose various gaits according to different conditions, which improves their adaptability [33]. Through observation, in most cases, the movement of an ant is realized by swinging two group legs (Group I: right front leg (RF), left middle leg (LM), right hind leg (RH); Group II: left front leg (LF), right middle leg (RM), left hind leg (LH).) alternately. This tripod gait is very efficient. In some special occasions such as uneven ground, an ant will swing its six legs in an orderly way to remain stable. Sometimes, the movement of an ant will be irregular, however, we found that regardless of gait, the trajectories of each foot tip was the same, but the phases were different. These abundant gait patterns of ants have been of great help in studying the locomotion of legged robots [34].

**Figure 1:**
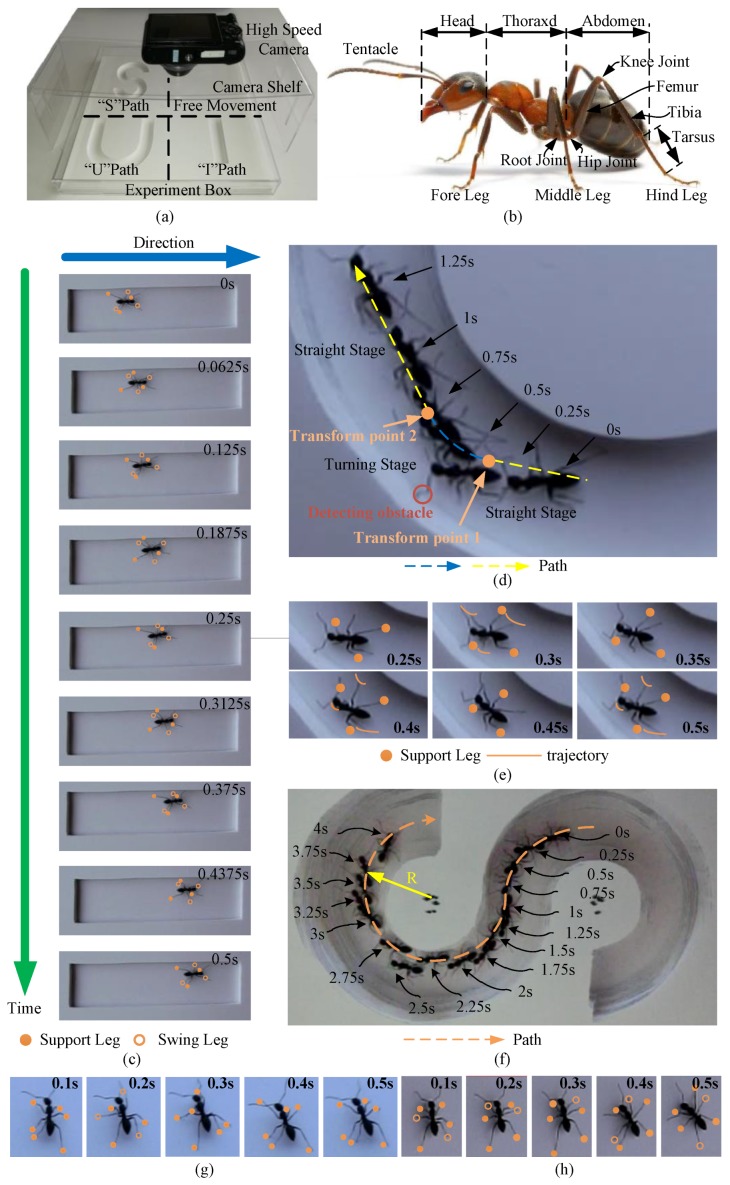
Ant experiments. (**a**) Experiment tools; (**b**) *Camponotus japonica* ants; (**c**) “I” path: The leg with the solid point represents the stance phase and the hollow circle represents the swing phase; (**d**) “U” path: dashed lines represent the path of movement; (**e**) Foot tip trajectory during turning: the curves represent the trajectory of the foot tip, and the point represents the supporting leg; (**f**) “S” path: the dashed curves with arrows indicate the advance direction; (**g**) Tripod gait: the leg with the solid point represents the stance phase and the hollow circle represents the swing phase; (**h**) Quadruped gait: the leg with the solid point represents the stance phase and the hollow circle represents the swing phase.

An ant experiment platform was carefully established and experiments planned for studying ant movement. The platform is shown in Figure 1a, and consisted of a high-speed camera, camera shelf, experiment box, and PVC board. Both the experiment box and camera shelf were made of transparent acrylic board so that it had little influence on the photography. The PVC board was placed in the experiment box and divided into four parts (“I” path for straight gait, “U” path for switching between straight and turning, “S” path for always turning and switching direction of turn, and free movement area). The ant moved in these paths and the gait and trajectory observed. To obtain a clear photograph, the high-speed camera was set at 240 fps. A *Camponotus japonica* ant [35] was chosen for the experiments, and is shown in Figure 1b. This type of ant has a big trunk and long legs. The whole body is nearly 10 mm long, and the leg more than 5 mm. This made it convenient to observe, and this structure has a wide movement space and high athletic performance [36]. The results of the experiments are described in detail below.

#### 2.1.1. ‘I’ Path for Straight Gait

The ant in the “I” path is shown in Figure 1c. The shooting time was 0.5 s. According to the marks, the ant adapted a tripod gait in straight motion [37]. At 0 s, the Group I legs were in the swing phase, and the Group II were in the stance phase. At 0.0625 s, the states of these two group legs were reversed. At 0.125 s, they returned to their original state. According to this rule, the legs of the two Groups swung alternately and supported the body to move forward until 0.5 s.

#### 2.1.2. ‘U’ Path for Switching between Straight and Turning

The movement of ant in the “U” path is shown in Figure 1d. In the beginning, the ant kept moving in a straight line. At 0.25 s, the tentacle of the ant detected an obstacle and started to turn. Then, it changed to straight motion again at 0.5 s. This phenomenon was really interesting, so we slowed down the video between 0.25 s and 0.5 s to observe the trajectory of the foot tip during the moment, and captured six pictures, which are shown in Figure 1e. At 0.25 s, the legs of Group I were in the supporting state and turned to the swing state at the next moment. In contrast, the legs of Group II began to support the body. During 0.25–0.375 s, the legs of Group I began to change to the satisfied trajectory for turning, and this process ended before touching down. After transition, these legs began to support the body to make the trunk turn. During 0.375–0.5 s, like with the Group I legs, the legs of the Group II completed the transition in the swing state and drove the ant to turn after touching down. At this moment, the conversion process of the ant from straight to the turn was completed. The ant performed good stability through the short process since the trajectory always changed in the air. This is because only the leg in the support state affects movement, which why the locomotion of an insect can change smoothly.

#### 2.1.3. ‘S’ Path for Always Turning and Switching Direction of Turn

The movement of an ant in the “S” path is shown in Figure 1f. The whole running time was 4 s. The dashed line with the arrow represents the path and direction of the ant. In this process, the trajectory of the center of gravity (COG) coincided with the ideal path. In one gait period, the trunk of the ant will turn in a small angle. Therefore, the turning of an ant can be viewed as a superposition of multiple arcs and a repetition of a single gait.

#### 2.1.4. Free Movement

In the free movement area, the gait pattern of an ant is very manifold. The tripod gait is shown in Figure 1g. At 0.1 s, six legs were in the support state; at 0.2 s, the Group I legs were in the swing phase, and the Group II legs were in the stance phase; at 0.3 s, the movement form of the legs from both Groups began to interchange; at 0.4 s, the Group I legs were in the stance phase, and the Group II legs were in the swing phase; at 0.5 s, the legs returned to their initial state. Usually, ants move forward with a tripod gait, especially in fast motion. For six-legged insects, this gait is the most efficient and stable. Sometimes, an ant will choose a quadruped gait [38], which is shown in Figure 1h. At all times, at least four legs are in the support state, which provides great stability. Aside from their typical gaits, ants will use irregular gaits in special circumstances. These irregular gaits also play an important role in ant movement.

In short, ants use different movement patterns to cope with different environments. Especially in turning, ants can go through all kinds of crooked paths and will adjust their direction when an obstacle is detected. However, the key to the change of movement is the change of foot tip trajectory. That is to say, the movement of an ant is determined totally by the trajectory of the foot tip. According to the state of the foot, the trajectory can be divided into the stance phase and the swing phase. When the ant walks in a straight line, the swing trajectory is approximately an arc. This arc is linked with the trajectory of the stance phase and forms a closed loop. The support trajectory is approximately a straight-line relative to the body, or can be considered as an arc with a large radius. The turning movement of an ant is also very flexible. The swinging trajectory is an arc, but the support trajectory becomes a curve in the horizontal plane [39]. In the supporting trajectory, the inner side of the turn has a larger curvature than that of the outside. The legs in the stance phase provide power for moving and control the direction of the ant. As for robots, the foot trajectory for straight and turning can also be planned as per the path of the ant. As long as the curvature of the trajectory is regulated according to requirement, the robot can perform different movements.

### 2.2. Material and Platform

**Figure 2:**
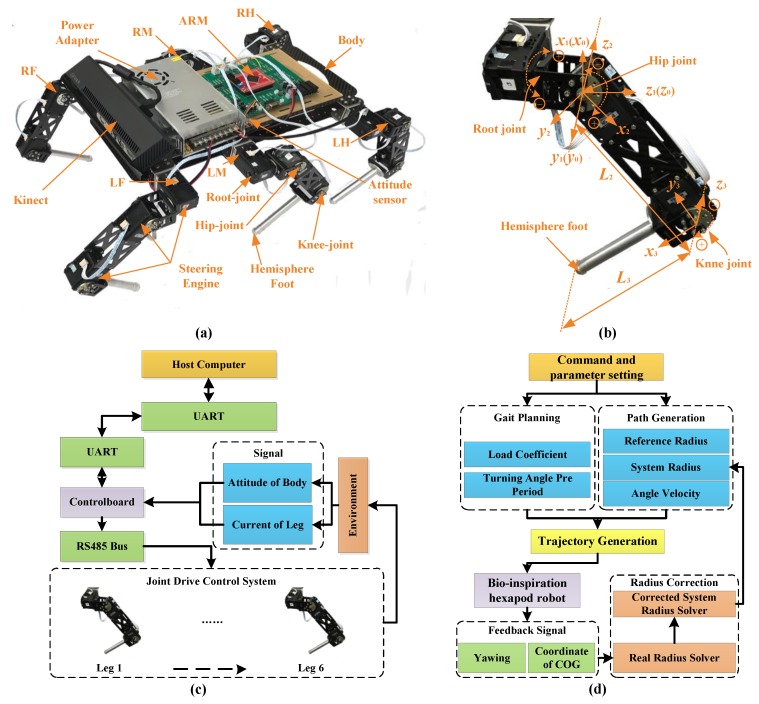
(**a**) Biomimetic hexapod robot; (**b**) Leg structure parameters; (**c**) Control diagram; (**d**) Algorithm framework.

The SmartHex experimental robot platform is shown in Figure 2a. The robot adopts a bionic structure and lightweight design principle. The six legs were divided into two groups and were distributed to the side of the robot trunk, respectively [40]. The total weight was 5.4 Kg. The shank, foot, and connector of the digital motor were all made with an aluminum alloy. The trunk was made out of hollowed-out carbon fiber. Each leg had three rotator joints. The parameters of each leg are shown in Figure 2b. The torque of each joint was provided by a smart motor. The rotation axis of the root joint was parallel to the forward direction of the robot, which provided lateral support force. The rotation axis of the hip joint and knee joint were perpendicular to the root joint, which provided power for moving forward [41]. The robot was equipped with a Kinect camera (Microsoft, Redmond, WA, USA), a power module, a control panel, and a sensor system [42]. Kinect cameras were used to detect obstacles and terrain recognition. The power module provided power for the entire robot. The control board was used for communication between the host computer and the digital servo system. The sensor system was used to acquire attitude signals and current signals. The hardware architecture of the robot is shown in Figure 2c. It was mainly composed of the host computer, lower computer, and digital servo motors. The computation of the algorithm was done in the host computer and obtained the motion data of each leg. Then, these data were transmitted to the Cortex-M4 control board (ARM, Cambridge, UK). After processing, the data were used for driving the motions. The signal acquisition was mainly dependent on the current detection units and the attitude sensor. These feedback signals were sent back to the host computer for detecting the movement condition of the robot.

The framework of the control strategy is shown in Figure 2d. The whole system was composed of a command and parameter setting, gait planning, path generation, foot trajectory generation, experimental prototype, feedback signal, and a radius correction algorithm. The command provided the input for the control system. Gait planning was controlled by the gait parameters and turning angle in a period. Additionally, path generation was controlled by the reference radius, system radius, and turning angle velocity. At the beginning of correction, the system radius equaled the reference radius. The robot then adjusted the system radius to eliminate the deviation. When the deviation was 0, the system radius converged to a value. It was regarded that the system radius was a parameter that drove the robot to obtain the desired real radius. Together, the gait planning module and the path generation module generated the trajectory of the foot tip. Through the inverse kinematics, the control signals of each leg were obtained to drive the robot. The feedback signals of the yaw angle and COG coordinates were transmitted to the radius correction algorithm module and system radius was calculated. The whole algorithm architecture was in closed loop control and could achieve different movement forms according to the parameters.

### 2.3. Extended Turning Strategy

During turning, the trajectory of the COG must coincide with the desired trajectory. Regardless of the kind of gait used, the trajectory of the COG is made up of many small arcs of a gait cycle [43]. The center angle of an arc was defined as gait angle *θ*. The turning process was regarded as a repeat of a single gait cycle. Therefore, taking a gait cycle as an example, the strategy of turning was analyzed.

**Figure 3:**
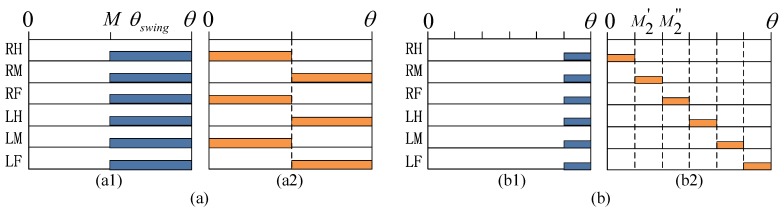
Gait pattern of turning (**a**) Tripod gait; (**b**) Wave gait.

The gait pattern of turning can be described by Figure 3 where the abscissa represents the gait angle, and the ordinate represents the number of legs. The colored rectangle represents the position of the swing phase in a gait cycle. The blank represents the leg in the stance phase. We assumed that the swing phases of each leg were all in the last section of a gait cycle at the beginning of planning, which is shown in Figure 3(a1). The angle *θ_swing_* and the starting point *M* of the swing phase can be expressed as:

(1) $$\theta_{swing}=(1-\beta )\theta$$

(2) $$M=\theta -\theta_{swing}$$

where *β* is the duty factor. When the robot turns in tripod gait, the gait chart is shown in Figure 3b. The change from Figure 3(a1,a2) can be considered as the phase of swing moved to a special position. In this way, the planning of wave gait can be represented by Figure 3b. After moving, the start angle *M_i_’* and end angle *M_i_’’* of the swing phase of each leg are as follows:

(3) $$M_{i}{}^{\prime }=\{\theta_{swing}(i-1)-fix[\theta_{swing}(i-1)]\}\theta$$

(4) $$M_{i}{}^{\prime \prime }=M_{i}{}^{\prime }+\theta_{swing}=\{i(1-\beta )-fix[(i-1)(1-\beta )]\}\theta$$

where *i* represents the number of legs, *θ* represents gait angle; and *fix*[] represents the integer operation. It is known that regardless of gait, the phase of swing state of any leg can be described by Equations (3) and (4).

As is well-known, the turning of a robot is realized during the stance phase. When the leg lifts off the ground, it does not have any impact on the movement of the robot. Only when the leg touches the ground, can the movement of robot be realized via by the moving of the leg. When the leg is in the stance phase during the movement, the foot is stationary relative to the earth coordinate system, and it is mobile in the COG coordinate system. The stance trajectories of each leg for turning are calculated by the relative motion between the COG and foot as it is not an arc of a cycle, but curves. The world coordinate is established at the center of the turning path, and uses it as a reference coordinate. When the robot moves, the path of COG coordinate is an arc with a radius of *R* in the world coordinate, and the positive direction of the *X* axis always points to the center of the arc. When the body moves into a random middle position *B_δ_* of a gait period, according to the coordinate transformation, the coordinates of the foot tip PAiBδ in the coordinate system *B_δ_* are as follows:

(5) PAiBδ=RBIBδPAiBI+PBIORGBδ=(XAiBIcosωt+YAiBIsinωt−R[1−cosωt]−XAiBIsinωt+YAiBIcosωt−RsinωtZAiBI)

where RBIBδ is the rotation operator from initial coordinate *B_I_* to initial coordinate *B_δ_* of a gait period; PBIORGBδ is the translation operator from *B_I_* to *B_δ_*; *ω* is the turning angular velocity; (XAiBI,YAiBI,ZAiBI) is the coordinate of the foot tip in the *B_I_* coordinate; *T* is the gait period; and the range of *t* is [0 ≤ *t* ≤ (1-*β*)*T*].

To ensure that there is no impact and smooth transition can be maintained between the foot tip and ground when they come into contact or separation, the quartic polynomial is adopted to plan the trajectory of the swing phase [44]. The function of the swing trajectory is:

(6) $$\{\begin{array}{c} x=a_{0x}+a_{1x}t+a_{2x}t^{2}+a_{3x}t^{3}+a_{4x}t^{4}\\ y=a_{0y}+a_{1y}t+a_{2y}t^{2}+a_{3y}t^{3}+a_{4y}t^{4}\\ z=a_{0z}+a_{1z}t+a_{2z}t^{2}+a_{3z}t^{3}+a_{4z}t^{4} \end{array}$$

The coefficients of each component are:

(7) $$A={\left[T\right]}^{-1}\cdot \left[X\right]$$

where [*T*] is the time matrix; and [*X*] is the coordinate matrix.

When the radius parameter *R* in the turning algorithm is large, the turning can be extended to the straight line. However, when *R* is too large, the arc length of a gait angle *θ* will become larger, that is, the path of the robot’s COG in a gait cycle will be longer. Nevertheless, due to the constraints of leg geometry, the robot may not be able to reach when *R* is very large. Therefore, it is necessary to calculate the maximum turning angle of the robot in a gait period.

When the robot turns to the end point of the stance phase *θ_i_^s^*, the position of the foot tip in the COG coordinate is:

(8) PAiBS=(XAiBSYAiBSZAiBS)=(XAiBIcosθis+YAiBIsinθis−R[1−cosθis]−XAiBIsinθis+YAiBIcosθis−RsinθisZAiBI)

where *R* is the turning radius. When the root joint, knee joint, and foot tip can form a triangle, that means that the leg satisfies the geometric constraint. The geometric constraint equations of each leg can be expressed as:

(9) ‖PAiBS−PO1,iB‖≤L2+L3

where POiB is the position of the root joint under the COG coordinate. *L_2_* and *L_3_* represent the length of thigh and shank, respectively. Maximum turning angle in one gait cycle is:

(10) $$\theta_{\max}=\max\{\frac{\theta_{i}^{s}}{\beta }\}(i=1,2,3,4,5,6)$$

According to the analysis above, as long as the radius is changed, the transition between the turning and straight can be achieved. However, it will cause irregularity in the signal of each joint when the motion parameters are changed suddenly. At the same time, that will lead to discontinuity in the foot trajectory and reduce stability. If the strategy of the transition radius is adopted slowly, although the influence on stability is reduced, the transition time is too long, which is not good for the robot’s speed of response. Therefore, a transition strategy that satisfies rapidity and stability was proposed. By imitating the switch method of an ant, as long as the moment of switch is controlled in the swing phase and finished before touching down, the switch could be achieved instantaneously and guarantee stability.

Each leg of the robot was designed to be controlled independently. After receiving the turn signal, the movements of each leg were detected respectively. If the foot was in the stance phase at that moment, the radius remained unchanged. It changed instantaneously until it entered the swing phase and reached the highest point of the trajectory. In another case, if the foot was in the swing phase at that moment, similarly, the radius remained unchanged. It changed instantaneously until it entered the swing phase again and reached the highest point. Each leg was controlled according to the method above, and they did not affect each other. The pseudo code of the extended turning algorithm is shown in Algorithm 1.

**Algorithm 1:** Extended turning algorithm. **Initialize:** the length of thigh *L*_1_, the length of shank *L*_2_, gait coefficient *β*, radius of turning *R*, angle velocity *ω*, *Flag_change_* = 0, *Flag_swing_* = 0.**Initial movement form:**  (1) Movement form change detection  **If** (*Flag_change_* = 1)    (2) Calculating the maximum turning angle in one gait cycle *θ*_max_    (3) Planning the gait of robot. Generating start angle *M_i_’* and end angle *M_i_’’* in swing phase of each leg    (4) Generating the trajectory function of foot tip based on quartic polynomial    (5) Generating the control signal of each joint according to inverse kinematics      (6) Leg state detection      **If** (*Flag_swing_* = 1)       (7) Change the corresponding parameters of the leg      **else**       (8) Jump to (6)      **end**  **else**    Repeat the initial movement form and jump to (1)  **end****Until:** The change of parameters is completed and new movement is formed.

### 2.4. Radius Correction Algorithm

**Figure 4:**
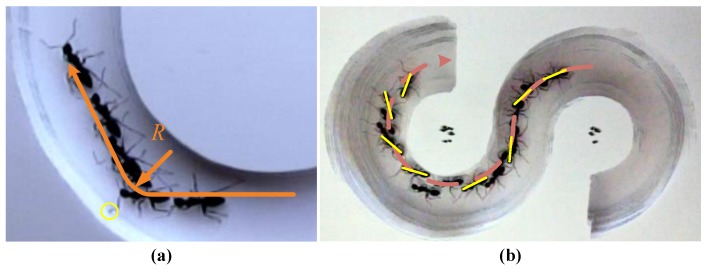
Bio-inspiration of radius correction. Movement of ant in “U” path (**a**) and in “S” path (**b**).

Straight motion is the simplest and most efficient form of movement for ants. Through our experiments, we found that regardless of the situation, an ant will first choose straight motion. The ant in the “U” path is shown in Figure 4a. Initially, the ant moved straight, which can be regarded as a turning with infinite radius. The yellow circle indicated that the tentacle of the ant had detected an obstacle and had momentarily deviated from the desired trajectory. Then, the ant began to adjust its turning radius. The radius adjustment of the ant in the “S” path is shown in Figure 4b where the dashed line represents the path and the yellow lines represent the body direction. It can be concluded that the ant was constantly adjusting its yaw angle during turning. In other words, it kept adjusting its radius as much as possible for the body to move along its trajectory. Therefore, the path correction of an ant is completed by adjusting the radius.

Due to foot tip slippage, the real turning trajectory of a robot will deviate from the desired trajectory just like an ant. Thus, the trajectory of a robot can be corrected much like an ant. Therefore, the path correction of the robot in this paper was implemented by modifying the radius in real-time.

**Figure 5:**
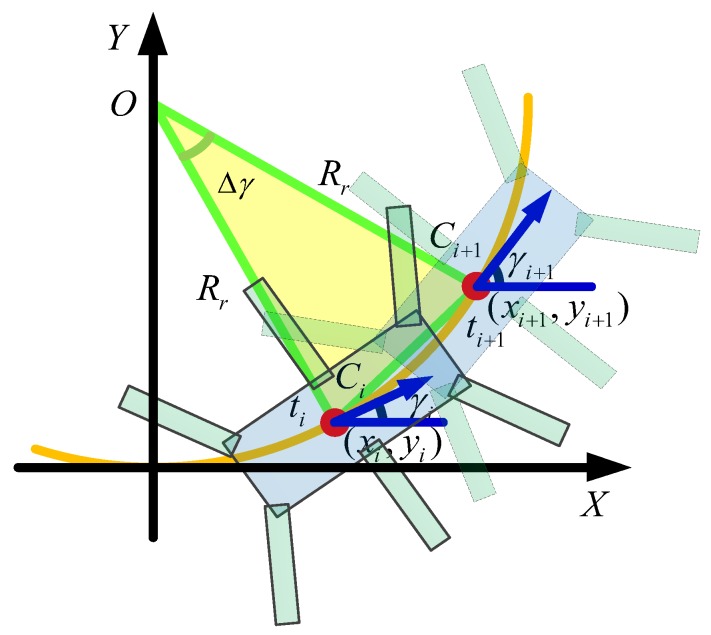
The solution of real radius correction.

A sketch of the radius calculation is shown in Figure 5. Supposing that the robot goes to the *C_i_* point at the time of *t_i_*_,_ and reaches the *C_i+_*_1_ point at the time of *t_i+_*_1_. The feedback signals at the time of *t_i_* and *t_i+1_* are the yaw angle (*γ_i_*, *γ_i+_*_1_) and COG coordinate (*C_i_ =* (*x_i_,y_i_*), *C_i+_*_1_ *=* (*x_i+_*_1_*,y_i+_*_1_)), respectively. An isosceles triangle was developed by constructing auxiliary lines through the two points *C_i_ C_i_*_+1_ and was perpendicular to their direction of yaw. The two auxiliary lines intersected at the *O* point. The line *OC_i_* was the real radius in this period. According to the cosine theorem, the real turning radius $R_{r}^{\left(i\right)}$ is:

(11) $$R_{r}^{\left(i\right)}=\sqrt{\frac{{\left(x_{i+1}-x_{i}\right)}^{2}+{\left(y_{i+1}-y_{i}\right)}^{2}}{2[1-\cos(\gamma_{i+1}-\gamma_{i})]}}$$

There is a proportional relationship between the system radius and the real radius, that is:

(12) $$R_{s}\propto R_{r}$$

The system radius is a parameter in the program. Therefore, the corrected system radius is:

(13) $$R_{s}^{\left(i\right)}=\frac{R_{s}^{\left(i-1\right)}R_{ref}}{R_{r}^{\left(i\right)}}\;\left(i=1,2,3\ldots \right)$$

where $R_{r}^{\left(i\right)}$ is the real radius in the *i*th feedback; $R_{s}^{\left(i\right)}$ is the system radius in the *i*th feedback; $R_{ref}$ is the reference radius of our expectation; and initialization of system radius is $R_{s}^{\left(0\right)}=R_{ref}$.

Correction error of the system radius in *i*th feedback is:

(14) $$E_{i}=R_{s}^{\left(i\right)}-R_{s}^{\left(i-1\right)}$$

This algorithm requires the robot to acquire the COG coordinate and the yaw angle parameter during the movement. It needs to establish a running environment map model and positioning system, therefore, it is impractical for a robot in a strange environment and will reduce the generality. When the error is stable at zero, the system radius will converge to be a constant. We can take the converged system radius directly as the system radius of the robot motion. Hence, a simplified algorithm for radius correction was proposed based on this. Since there is a certain relationship between the reference radius and system radius, the focus of the simplified algorithm as to find this relationship by fitting in order to obtain a more accurate system radius.

After *k* periods, the system radius stabilizes. The system radius from the *k*th to (*k + n*)th periods is collected and the average is:

(15) R¯s(Rref,β)=∑kk+nRs(i)(Rref,β)(k−i)

where Rs(i)(Rref,β) is the system radius under duty factor *β* and reference radius *R_ref_* at the *i*th moment; R¯s(Rref,β) is the average system radius under duty factor *β* and reference radius *R_ref_*. In this way, the average system radius under different duty factors and reference radiuses can be worked out.

**Figure 6:**
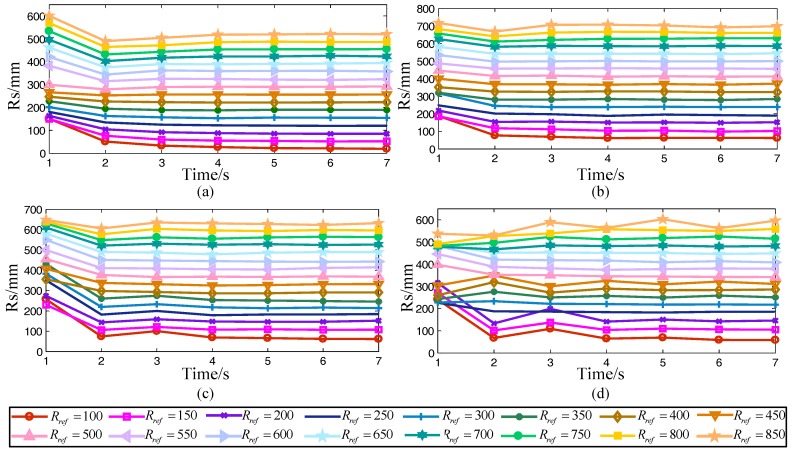
The curves of system radius under different theoretical radius and duty factor during the correction. (**a**) *β* = ½; (**b**) *β* = ¾; (**c**) *β* = 4/5; (**d**) *β* = 5/6.

The curves of the system radius under different reference radiuses and duty factors (*β =* 1/2, *β =* 3/4, *β =* 4/5, and *β =* 5/6) during the correction are shown in Figure 6.

**Figure 7:**
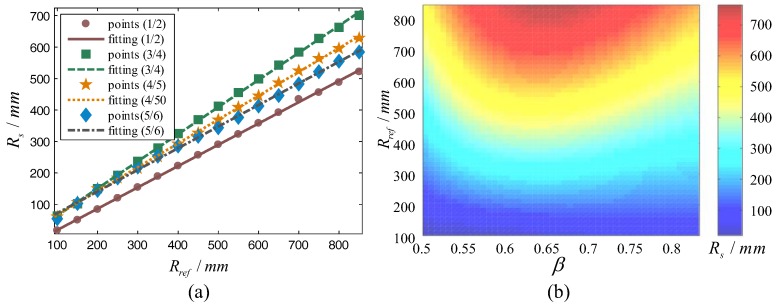
(**a**) Fitting curves; (**b**) Radius correction fitting surface.

Regardless of the duty factor and the reference radius, after correction, the system radius will eventually converge at a certain value. Taking the data after stabilization and calculating their average, the average system radius was obtained. After that, taking the average system radius as the abscissa and the corresponding reference radius as the ordinate and fitting them together, the fitting curve is shown in Figure 7a. The equation is:

(16) $$R_{s}=aR_{ref}+b$$

The parameters of Equation (16) are shown in Table 1.

In the previous section, only the radius correction curves of several typical gaits were fitted. As the duty factor and the reference radius both increase linearly, we used the above data to fit the surface to obtain the system radius under an arbitrary reference radius and gait where the X axis is duty factor *β*, the Y axis is the reference radius *R_t_*, and the Z axis is the system radius *R_s_*. When 0.5 < *β* < 3/4, the phenomenon that two front legs or two hind legs lift together appeared. This state for a hexapod robot is likely to cause a COG beyond the support polygon and lead to collapse. Therefore, a duty factor in the range of 0.5 < *β* < 3/4 was not recommended and was excluded in the fitting. To improve the fitting accuracy, polynomial equation fitting was adopted. The fitting surface is shown in Figure 7b. The parameters of the surface equation are shown in Table 2.

The surface equation is:

(17) $$\begin{array}{ll} R_{s} & =p_{00}+p_{10}x+p_{01}y+p_{20}x^{2}+p_{11}xy+p_{02}y^{2}+p_{30}x^{3}+p_{21}x^{2}y+p_{12}xy^{2}+p_{03}y^{3}\\ & +p_{31}x^{3}y+p_{22}x^{2}y^{2}+p_{13}xy^{3}+p_{04}y^{4}+p_{32}x^{3}y^{2}+p_{23}x^{2}y^{3}+p_{14}xy^{4}+p_{05}y^{5} \end{array}$$

The whole radius correction algorithm can be summarized by the pseudo code, which is shown in Algorithm 2.

**Algorithm 2:** Radius correction algorithm.**Initialize:** coordinate of COG *P*; yaw angle *γ*; reference radius *R_ref_*; and system radius *R_s_*: *R_ref_ = R_s_*; sample period *T*; duty factor *β*; minimum duty factor *β_min_*; maximum duty factor *β_min_*; minimum reference radius *R_ref_*_,*min*_; maximum reference radius *R_ref_*_,*max*_; **for** (*β = β_min_*; *β < β_max_*; *β++*)  **for** (*R_ref_ = R_ref_*_,*min*_; *R_ref_ < R_ref_*_,*max*_; *R_ref_++*)    **Repeat:**    (1) Input *R_s_* and *β*
    (2) when *i*th sample period , collect feedback signals *P_i_* and *γ_i_*    (3) when (*i*+1)th sample period, collect feedback signals *P_i_*_+1_ and *γ_i+_*_1_    (4) Calculate real radius $R_{r}^{\left(i\right)}$ in *i*th correction period     (5) Input $R_{r}^{\left(i\right)}$ to the system radius corrector and output corrected system radius $R_{s}^{\left(i\right)}$    (6) Replace the system radius with the corrected system radius    **Until:** The radius error are eliminated and record the system radius Rs(Rref,β) under the *R_ref_* and *β* of this cycle  **end****end**  (8) Fitting the Rs(Rref,β) under different *R_ref_* and *β*, the radius correction surface equation is obtained**Result:** The radius correction surface equation can correct radius error directly.

## 3. Simulations

### 3.1. Straight-Turning Transition Simulation

**Figure 8:**
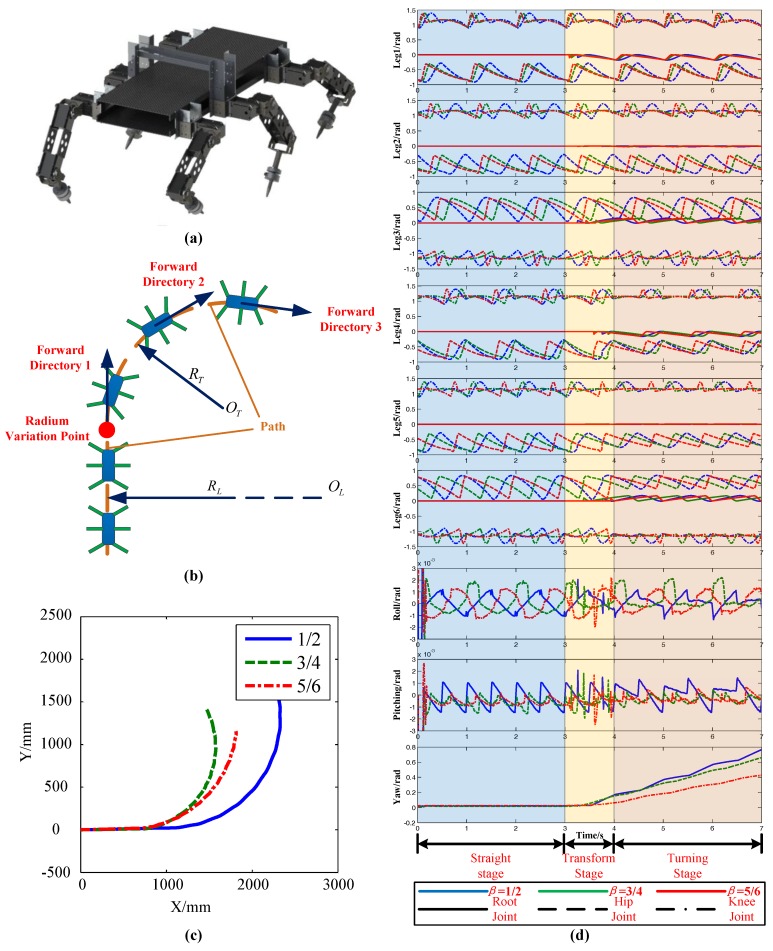
Simulations without correction. (**a**) Visual prototype; (**b**) Transition from straight line to turning; (**c**) The COG Path of different gaits changing duty factor; (**d**) Joint angle of each leg and altitude angle.

In this section, the transition of straight to turning was simulated to verify the performance of the extended turning algorithm transition and its influence on stability. The virtual prototype is shown in Figure 8a. Supposing that the robot first moved in a straight line. At the turning point, the robot starts to turn at radius *R_T_*. The diagram is shown in Figure 8b. The simulation parameters are: radius in straight *R_L_* = *inf*, radius in turning *R_T_* = 800 mm, gait period *T*_gait_ = 1 s, time consumption in transition *T*_transition_ = 1 s, body height H = 250 mm, lift height of leg *h* = 30 mm, duty factor *β* = 1/2, *β* = 3/4, and *β* = 5/6. The COG path under different gaits during the movement is shown in Figure 8c. It can be concluded that the extended turning algorithm proposed in this paper could make the robot transform from straight motion to turning motion in an arbitrary gait, but the robot had poor tracking ability for path. However, this was improved after the radius correction algorithm was implemented.

The curves of each joint angle and attitude under different gaits are shown in Figure 8d. Before 3 s, the root joints of each leg remained still, which meant that the robot kept going straight. At the stage of 3 s to 4 s, the root joints began to rotate in succession and the robot began to switch from straight to turning. After 4 s, the robot was completely in turning form. The curves of each joint indicated that the simulation experiment satisfied the expected motion hypothesis.

From the attitude analysis, the yaw angle in the first 3 s was stable around 0, this time, however, the robot was in straight motion. After 3 s, the yaw angle started to increase linearly. At this stage, the robot was turning and this result also compounded the expected parameters of the simulation. There was a larger fluctuation of pitch at 0.3 s before the robot started, which was caused by the initial pose of the robot. However, when the robot started to move, the pitch angle stabilized around 0. Furthermore, the second big wave of the robot’s pitch angle occurred at 3 s, which was caused by the switch between straight and turn. After about 1 s, the pitch angle at 4 s stabilized and the robot switched over. Although there was a slight fluctuation in the pitch angle of the robot during the transition, the maximum fluctuation range was only ±0.002 road, which had no effect on the motion of the robot. For the roll angle, the change was similar to the pitch angle. It can be concluded that the extended turning algorithm was effective regardless of the gait of the robot, and could maintain good stability during the transition.

### 3.2. Radius Correction Simulation

**Figure 9:**
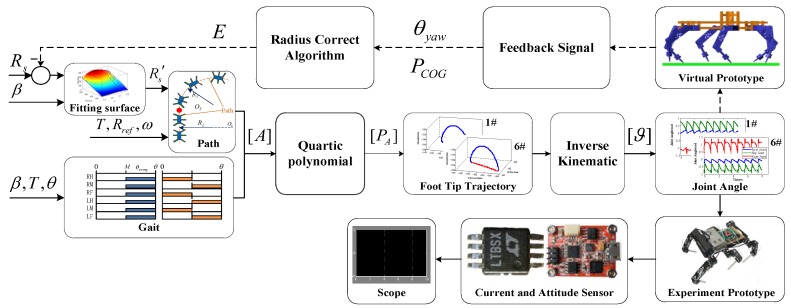
Simulation framework.

On the virtual prototype platform, once the feedback signal was added to the original kinematics algorithm, a closed-loop control was established. The framework of the simulation is shown in Figure 9, which was composed of parameter input, kinematics control, virtual prototype, feedback signal, and the radius correction algorithm. System parameters included clock signal *T*, gait *β*, system radius *R*_s_, reference radius *R_ref_*, angular velocity *ω*, and gait angle *θ*, which provided the data for the whole system. Path planning and gait generation were completed in the kinematics model and coefficient matrix [A] of the foot trajectory curve equation, and foot coordinate [*P*_A_] were generated next. After inverse kinematics, the drive signals [*ϑ*] of each joint were produced and input to the prototype. During simulation, the yaw angle *θ_yaw_* and COG coordinate *P*_COG_ were output as the feedback signal. The radius correction module obtained the feedback signal and then reprogrammed the system radius. After the simulations, the radius corrected surface equation was generated. For the experimental platform, the corrected system radius was obtained directly through the correction equation. The current of the legs and attitude data were collected by the corresponding sensors and transmitted to the host computer for observation.

**Figure 10:**
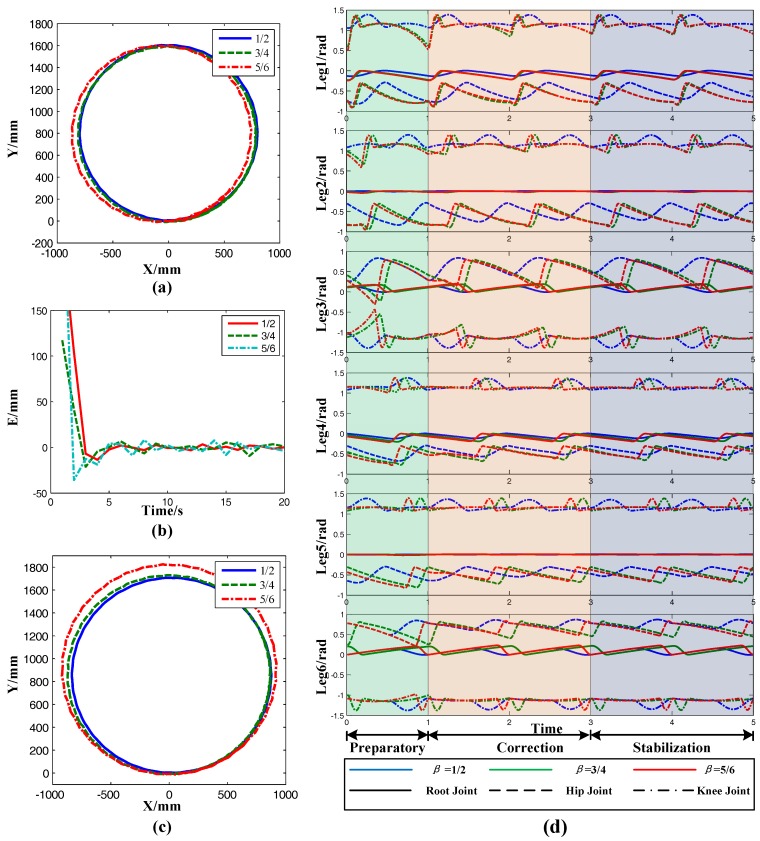
(**a**) Simulation path of the robot with radius correction algorithm; (**b**) Radius error curve during the correction; (**c**) Simulation path of the robot with radius correction surface function; (**d**) Joint angle of each leg.

The simulation paths of the COG with the radius correction algorithm are shown in Figure 10a where the reference radius was 800 mm and the duty factors were 1/2, 3/4, and 5/6, respectively. After introducing the feedback control, the robot had the ability to detect the radius. When the deviation was detected, the radius was automatically corrected so that the real radius approached the reference radius gradually. The error curves of the simulation are shown in Figure 10b. The error of the system radius was corrected constantly and eventually fluctuated around zero. In addition, the correction algorithm was very fast, and the error was eliminated at the end of the fifth gait period. The performance of the simplified radius correction algorithm is shown in Figure 10c. The parameter was the same as before. The radius of COG trajectory was almost 800 mm. Compared with the radius correction algorithm, the real radius obtained by the simplified correction algorithm was slightly larger than the reference radius, which was caused by the surface fitting error. However, the radius error did not much affect the actual motion of the robot. The variation of each joint during the radius correction is shown in Figure 10d. The COG and yaw angle signals were collected at 0 s and 1 s, so the radius will not be corrected at this stage. After 1 s, the real radius was calculated according to the data obtained by the two feedbacks. At this moment, the radius started to correct and the curves of each joint changed. After 3 s, the joint curve showed a new trend and did not change further, which showed that the radius error had been eliminated. The variation of the joint curve also satisfied the expected setting of the radius correction algorithm.

## 4. Experiments

To verify the correctness of the algorithm in this research, a series of experiments was conducted using the hexapod robot platform. There were three groups of experiments including movement transition, contrast of radius correction, and turning.

The outstanding feature of the radius correction algorithm is that it can be applied to any possible gait, so we selected two typical gaits and an arbitrary transition gait for testing. Meanwhile, the attitude data and current data were collected to judge the performance quantitatively. Experiment parameters are shown in Table 3.

**Figure 11:**
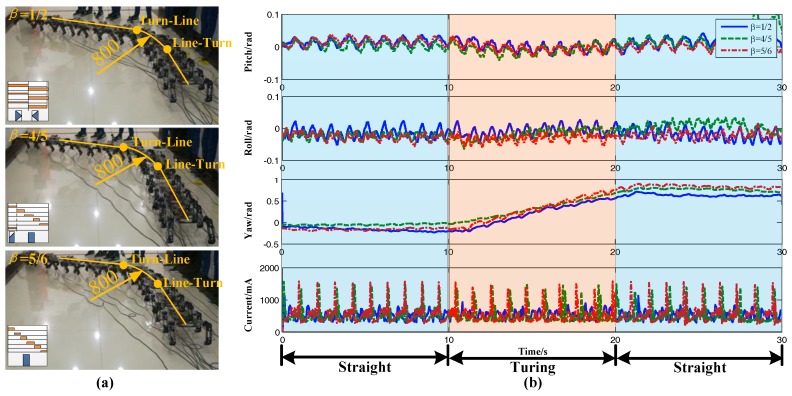
Straight-turning-straight experiment. (**a**) Image of different gait; (**b**) Attitude data and current data.

The graphs of straight-turning-straight under three gaits (tripod gait *β* = 1/2, transition gait *β* = 4/5, and wave gait [45] *β* = 5/6) are shown in Figure 11a. In the image, the floor tile is square and its edge length is 800 mm. Using the size of the floor tile as a reference, the robot could keep its direction in the straight line well. The transition between the straight and turning was smooth and the turning radius was about 800 mm, which satisfied the preset parameter condition. Figure 11b shows the corresponding attitude data and current data. The range of the pitch angle and roll angle were all less than ±0.2 rad. There was no obvious fluctuation during the transition at 10 s and 20 s. According to the curves of the yaw angle, from 0–10 s, the yaw angle was approximately horizontal and the robot moved straight. From 10–20 s, the yaw angle was a slash and the robot turned. From 20–30 s, the robot moved straight again. This fit perfectly with the preset motion parameters. Therefore, the extended turning algorithm could achieve a fast transition in different motion states and had good stability. The period of the current curve was 1s, and each period had a peak value that was equal to the gait period.

**Figure 12:**
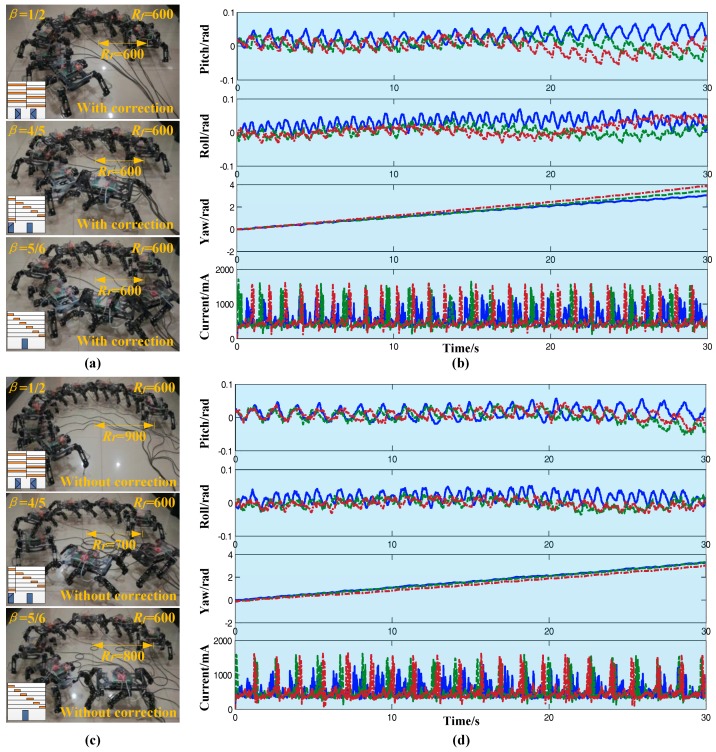
Comparison experiments on radius correction algorithm (reference radius *R_ref_* = 600 mm). (**a**) Images of different gaits (radius correction); (**b**) Attitude data and current data (radius correction); (**c**) Images of different gaits (without radius correction); (**d**) Attitude data and current data (without radius correction).

A comparison test of the radius correction algorithm was carried out where the reference radius was *R_ref_* = 600 mm and duty factors were 1/2, 4/5, and 5/6, respectively. The graphs showing turning with the radius correction algorithm are presented in Figure 12a. Using the floor tile as a reference, it was concluded that the real radius was approximately the same as the reference radius. Figure 12c is the stack graph without the correction algorithm under the same conditions as Figure 12a, where it was obvious that there was a large error of the real radius and the reference radius. The corresponding attitude data and current data are shown in Figure 12b,d. The range of pitch angle and roll angle were less than ±0.1 rad. The variation of yaw angle was an oblique line. The robot roughly turned 4 rad in 30 s. This showed that the stability of the robot was not greatly affected by the radius correction algorithm. The period of the current curve was 1 s, which was identical to the period of gait. According to these curves, the radius correction algorithm could effectively correct the turning radius error, and had almost no influence on the stability and energy consumption of the robot.

**Figure 13:**
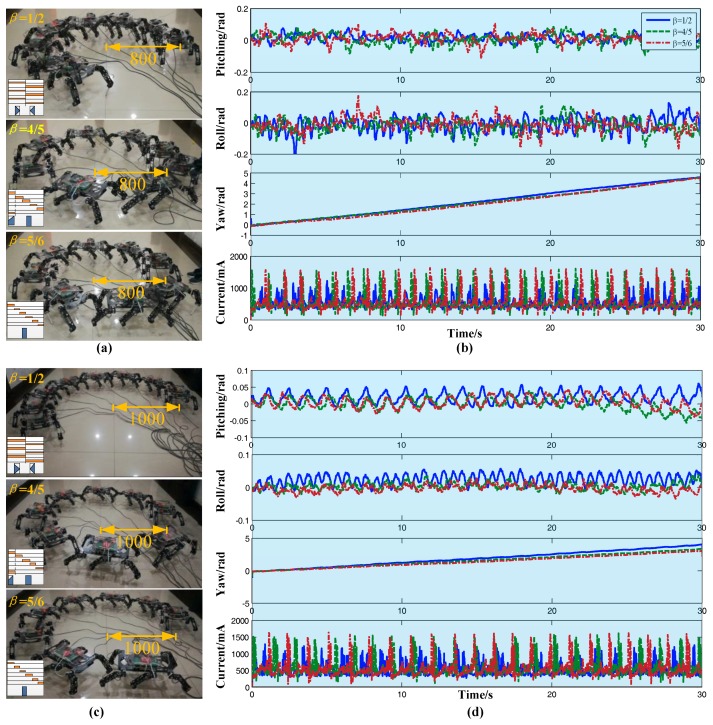
Turning experiment. (**a**) Images of different gaits (*R_ref_* = 800); (**b**) Attitude data and current data (*R_ref_* = 800); (**c**) Stack images of different gaits (*R_ref_* = 1000); (**d**) Attitude data and current data (*R_ref_* = 1000).

The correction ability of the algorithm for different reference radius was verified in this group of experiments. The three gaits above-mentioned were also selected here. We randomly chose two reference radiuses (*R_ref_* = 800 mm, *R_ref_* = 1000 mm) for the test, which are shown in Figure 13a,c, respectively. Figure 13b,d are the corresponding attitude angle curve and current curve, respectively. The robot still remained steady regardless of the radius. The correction algorithm showed good performance for both the arbitrary gait and the reference radius.

## 5. Discussion

In this paper, a turning control strategy and a radius correction algorithm for a hexapod bio-inspired robot were proposed based on the behavior of ants. In Section 1, the movement control of several typical legged robots [7,9,11,17,18,22] was described. In this section, we collected information on legged robots from the last ten years for comparison, which is shown in Table 4. The number of leg and gait patterns, movement form, degree of freedom of a single leg, and driving mode were selected as the comparison items. Normally, the more legs a robot has, the more difficult it is to control the gait. However, robots with multiple legs theoretically have better stability and load capacity [46,47] For robots with fewer legs such as biped robots [48,49,50], their motion is achieved by swinging one leg alternately. In most cases, it is balanced by one supporting leg. This kind of robot pays more attention to the control of motion stability.

From the research of legged robots from the last decade, the gait pattern of a quadruped robot includes walk [59], trot [60], pace [61], gallop [62], and bound [63], which are used for different conditions and are limited by the DOF (Degree of Freedom) of the leg and actuation of each joint. The first three types of gait are used for the slow movement of robots. The robot needs to adjust the position of its foot tip in real time to maintain its body balance [64,65,66]. The gallop and bound gaits can make the robot move quickly, but needs to build a complex kinetic model [67,68,69]. Hexapod robots also have a transition gait between typical gaits such as the tripod gait [70], quadruped gait [71], and wave gait [72]. Due to gait pattern diversity, the motion control of a hexapod robot is very difficult. However, on the basis of the previous studies, we proposed a simpler motion planning algorithm than previously described, which gave the robot the ability to achieve an arbitrary gait and greatly simplified the gait control method.

The movement forms of the robot mainly have a single form [51,55] and omnidirectional movement [41,52]. The control of the omnidirectional movement is more complex than the signal form. Most robots adopt different algorithms to achieve different movement forms [73,74,75]; however, this is not convenient for the control of the robot and will consume lots of energy. In this paper, the omnidirectional movement of the robot was achieved by extended the turning algorithm. The transition process of the different motion states was analyzed, which made it possible to transition instantaneously. Furthermore, we added the radius correction algorithm to the robot, which gave it the ability to correct its path and has not been mentioned in other works.

## 6. Conclusions

A common approach to planning the turning of a bio-inspired hexapod robot was raised, and inspired by biological behavior, a locomotion strategy for turning and deviation correction of a hexapod walking robot based on ant behavior was proposed. The movement experiments of ants under different conditions were studied using a high-speed camera, and the gaits and turning locomotion of the ants were analyzed. According to ant behavior, a turning strategy was proposed. On this basis, an extended turning algorithm and a method of smooth transition between the different radiuses were raised. Additionally, the most important proposal was that of a radius correction algorithm using the arbitrary gait of a bio-inspired robot based on bio-inspiration, which provided a method for robot self-correction. On the visual prototype, by taking the yaw angle and the coordinate of the COG as feedback signals, a closed-loop control was established. Based on the feedback signal, the real radius was obtained via geometric calculation and corrected effectively by adjusting the system radius. After that, a radius correction surface function was obtained, which was necessary for the robot to move in an outdoor environment without the positioning system and the environment model. This method simplified the control process greatly and enhanced the adaptability of the robot.

A series of simulations and experiments were carried out and the radius correction algorithm was verified on a hexapod walking robot prototype. Taking the tripod gait, transition gaits, and the wave gait as examples, the results of the experiments indicated that the turning algorithm could make the bio-inspired robot follow the desired turning path, and that the radius correction algorithm could reduce the error and make the robot follow the ideal path, regardless of gait or radius. Furthermore, the transition experiments between the turning and the straight indicated that the extended turning algorithm and the method of transition were correct. According to the data from the attitude sensor, it was concluded that the robot had good stability performance. Therefore, the ant-inspired turning strategy for a hexapod walking robot through the study of ant locomotion in this paper was effective.

## Figures and Tables

**Table 1:** Parameters of curve fitting.

**Coefficient**	**The Value of *β***			
	*β* = 1/2	*β* = 3/4	*β* = 4/5	*β* = 5/6
a	0.6772	0.8625	0.7577	0.6873
b	−49.12	−21.83	−9.204	3.769

**Table 2:** Parameters of surface fitting.

Parameters	Value	Parameters	Value	Parameters	Value
*p*_00_	4573	*p*_30_	−1.617 × 10^4^	*p*_13_	−2.87 × 10^−5^
*p*_10_	−2.15 × 10^4^	*p*_21_	−275.8	*p*_04_	3.206 × 10^−9^
*p*_01_	−39.86	*p*_12_	−0.1154	*p*_32_	−0.114
*p*_20_	3.26 × 10^4^	*p*_03_	6.231 × 10^−6^	*p*_23_	2.834 × 10^−5^
*p*_11_	184.9	*p*_31_	136	*p*_14_	−3.936 × 10^−9^
*p*_02_	0.02298	*p*_22_	0.1976	*p*_05_	−5.895 × 10^−13^

**Table 3:** Experiment parameters.

Parameters	Symbol	Value
Duty factor	*β*	1/2, 4/5, 5/6
Turning radius	*R_turning_*	600 mm, 800 mm, 1000 mm
Straight radius	*R_Straight_*	*Inf*
Running time	T	30 s
The moment of straight to turning	T*_S-T_*	10 s
The moment of turning to straight	T*_T-S_*	20 s
Gait period	T*_period_*	1 s

**Table 4:** Comparison between the recent multi-legged robots.

Robot (Year)	Leg Number	Gait Form	Movement Form	DOF/Leg	Actuation
Robot-EA308 (Erden et al. 2007) [51]	6	Free Gait	Going Straight	3	Servo Motors
BigDog (Raibert et al. 2008) [52]	4	Crawl Gait Trot Gait	Omnidirectional	4	Hydraulic Drive Linear Spring
SILO-6 (Estremera et al. 2010) [53]	6	free-crab gait		3	Servo Motors
COMET-IV (Irawan et al. 2012) [41]	6	Tripod Gait	Omnidirectional	4	Hydraulic Drive
HYQ (Boaventura et al. 2012) [54]	4	Cycle Gait	Trot Squat jump	3	Hydraulic Drive
Hexapod Robot (Jeong et al. 2013) [55]	6	Tripod Gait	Going Straight	3	Servo Motors
Cheetah-cub robot (Spröwitz et al. 2013) [56]	4	Trot Gait	Going Straight	2	Servo Motors with Cam Linear Spring Cable Mechanism
RHex-style hexapod robot (Chou et al. 2015) [57]	6	Leaping Running	Tripod Gait	1	Servo Motors Elastic Structure
Weaver (Bjelonic et al. 2016) [58]	6		Tripod Gait Wave Gait	5	Servo Motors
**Robot in this paper (2017)**	**6**	**Arbitrary gait (1/2 ≤ *β* ≤ 5/6)**	**Omnidirectional**	**3**	**Servo Motors**
